# Cleavages along {110} in bcc iron emit dislocations from the curved crack fronts

**DOI:** 10.1038/s41598-022-24357-5

**Published:** 2022-11-16

**Authors:** Tomoaki Suzudo, Ken-ichi Ebihara, Tomohito Tsuru, Hideki Mori

**Affiliations:** 1 Center for Computational Science and e-Systems, Japan Atomic Energy Agency, Tokai-mura, Ibaraki 319-1195 Japan; 2 Nuclear Science and Engineering Center, Japan Atomic Energy Agency, Tokai-mura, Ibaraki 319-1195 Japan; 3 Elements Strategy Initiative for Structural Materials (ESISM), Kyoto University, Yoshida, Honmachi, Sakyo-ku, Kyoto, 606-8501 Japan; 4 PRESTO, Japan Science and Technology Agency, Kawaguchi, Saitama 332-0012 Japan; 5 Department of Mechanical Engineering, College of Industrial Technology, Amagasaki, Hyogo 661-0047 Japan

**Keywords:** Structural materials, Theory and computation

## Abstract

Body-centered-cubic (bcc) transition metals, such as $\alpha$-Fe and W, cleave along the {100} plane, even though the surface energy is the lowest along the {110} plane. To unravel the mechanism of this odd response, large-scale atomistic simulations of curved cleavage cracks of $\alpha$-Fe were conducted in association with stress intensity factor analyses of straight crack fronts using an interatomic potential created by an artificial neural network technique. The study provides novel findings: Dislocations are emitted from the crack fronts along the {110} cleavage plane, and this phenomenon explains why the {100} plane can be the cleavage plane. However, the simple straight crack-front analyses did not yield the same conclusion. It is suggested that atomistic modeling, at sufficiently large scales to capture the inherent complexities of materials using highly accurate potentials, is necessary to correctly predict the mechanical strength. The method adopted in this study is generally applicable to the cleavage problem of bcc transition metals and alloys.

## Introduction

Body-centered-cubic (bcc) transition metals and alloys such as steel are ubiquitous in the infrastructure of automobiles, ships, bridges, and industrial plants such as nuclear power stations. It is widely known that materials with such a crystal structure are inherently brittle, and their resulting mechanical failures may have catastrophic consequences. To date, many fundamental investigations have been conducted on the brittle properties of these metals for e.g., tungsten^1–6^ and $\alpha$-Fe^7–18^.

Generally, precipitates, grain boundaries, and other lattice defects are intricately interrelated as causes of brittle fracture. Hence, the mechanism of brittle fracture is not fully understood and is therefore unpredictable. As discussed below, even the simplest kinds of brittle fractures without grain boundaries and precipitates cannot be modeled to sufficiently predict the preferential cleavage plane.

Theoretical investigations of brittle fractures are not a new subject: in the 1920s, Griffith^19^ established his theory that the energy release rate upon crack advance, *G*, must be equal to the energy of the two freshly exposed fracture surfaces: i.e., $G = 2\gamma _{S}$, where $\gamma _{S}$ is the surface energy. According to Griffith’s theory, the plane with the lowest surface energy is considered to be the preferential cleavage plane. It is known that the {100} and {110} planes have the lowest surface energy in bcc transition metals, with {110} having a slightly lower energy, while other crystal planes such as {111} have considerably higher surface energy^20–22^. Thus, the {110} plane should be the preferential cleavage plane. Nevertheless, it has been widely observed that cleavage in these metals occurs along {100}^20,23^. An exception is the case of embrittlement in martensitic steels, in which quasicleavage occurs along {110}^24,25^. This fact clearly indicates that the brittle cleavage fracture cannot be modeled by simple energetics; some unknown complex mechanism should be considered. To date, many studies attempted to solve this conundrum because it is expected that finding this missing piece can lead to a breakthrough to solve the complexity of fracture phenomena. Some authors considered plasticity to find a solution; for example, Ayres et al.^26^ claimed that plastic relaxation in the vicinity of the crack tip is responsible for the cleavage plane by the analyses of dislocation dynamics and predicted the preferential cleavage to be the {100} plane. Tyson^20^ claimed also that the plastic deformation near the crack tip on {100} is less than that on {110}. In contrast, Kohlhoff et al.^27^ claimed that each {100} crack plane provides two easy-propagating crack-front directions orthogonal to each other. Thus, an arbitrarily oriented crack front can always have a path along one or two easy-propagation directions. However, this is not the case for {110} planes. Kohlhoff et al.^27^ maintained that the preferential cleavage plane along {100} is not due to the plastic relaxation but due to this crack-propagation property of bcc transition metals. Riedle et al.^2^ conducted detailed analyses of single-crystal W fractures and supported the concept proposed by Kohlhoff et al., though they also suggested that plasticity is responsible for the preferential cleavage plane in the case of a high-temperature fracture. This historical dispute, whichever correct, implies that the absolute goal of brittle-fracture research is to correctly predict the brittleness and plasticity of every possible crack front direction.

**Figure 1:**
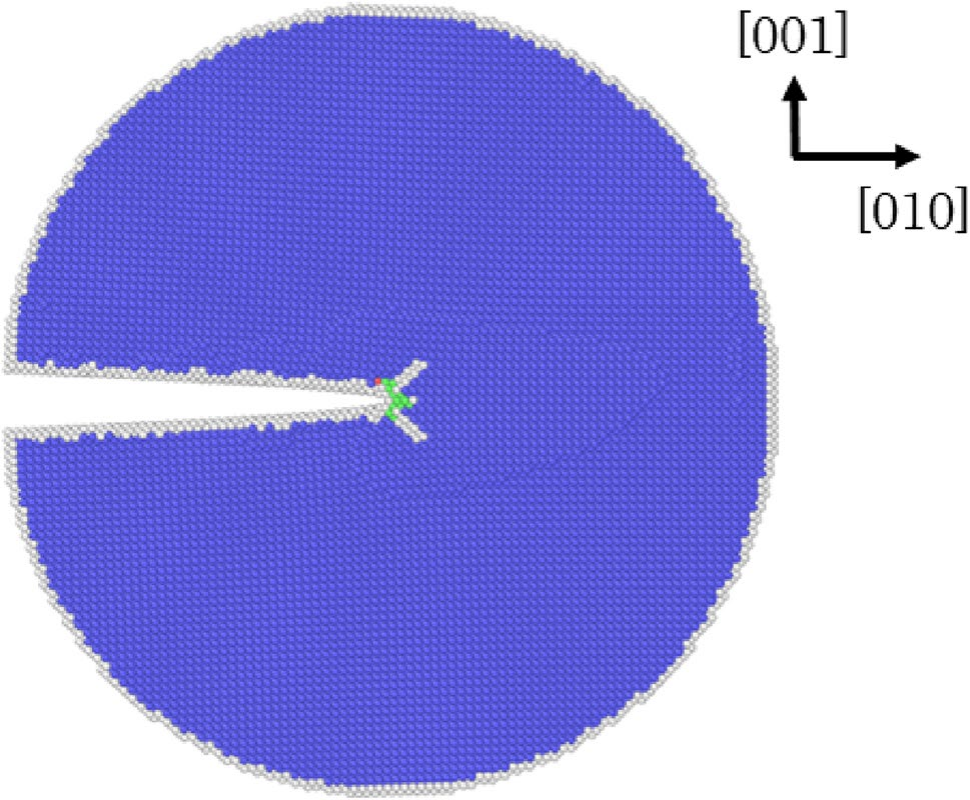
A numerical simulation of a straight crack front using an EAM potential^29^ shows typical artifacts, i.e. fictional plastic deformation at the crack tip. Coloring is based on common neighbor analysis; blue, green and white parts indicate bcc, fcc, and other crystals, respectively. Although the displacement field is not large enough for crack propagation, partial slips are observed along {110} planes in the vicinity of the crack tip, and an fcc crystal part appears as well.

Against this background, in our previous study^18^, we addressed this problem using large-scale three-dimensional (3D) molecular dynamics with embedded-atom-method (EAM) $\alpha$-Fe potential^28^ that reproduces a lower surface energy for {110} than that for {100}. In the previous study, mode-I loading of disk-shaped (or penny-shaped) cleavage cracks along different crystal planes was investigated: brittle fracture was observed with a modest amount of dislocation emissions from the crack tip of cleavage along {100}, while major plastic deformation relaxed the stress in the vicinity of the crack tip and suppressed the crack propagation along {110}. Accordingly, although the surface energy is not the lowest for {100}, the study successfully reproduced the preferential cleavage plane {100} and supported the claim that plastic relaxation at the crack tip on {110} is responsible for this plane not being the preferential cleavage plane. This success was, however, limited, because of the known drawbacks of EAM potentials. For example, they induce the inaccurate $\gamma$-surface (see Supplementary Fig. 1 for the details) that causes some artifacts (see Fig. 1) at crack tips^17^, which lead to fictional ductile behaviors instead of correct brittle behaviors, and it is also known that the fracture behaviors depend on the applied EAM potential^6^.

Recently, many empirical potentials were established using machine-learning technique. Examples of such potentials are the Gaussian approximation potential (GAP)^30^, spectral neighbor analysis potential^31^, and artificial neural networks (ANNs)^32^. These new types of potentials do not suffer from the aforementioned long-standing problem that affects EAM potentials. For example, Alam et al. created a new Mo potential using the ANN technique^33^, overcame the $\gamma$-surface problem, and suppressed the fictional plasticity at the crack tips. They also theoretically evaluated the critical stress intensity factors for dislocation emission ($K_{R}$) and that for crack propagation ($K_{G}$) for various straight crack fronts and found that $K_{G}<K_{R}$ for all the crack fronts, indicating that all the considered crack fronts are brittle. Their study did not support the fact that {100} is the preferential cleavage plane of bcc transition metals even though they applied an accurate machine-learning potential, suggesting that the stress intensity factor is not a sufficient measure for modeling the cleavage fractures of bcc transition metals.

With regard to Fe potential, Mori and Ozaki^34^ established an accurate empirical potential using the ANN technique and overcame many shortcomings of the previous EAM potentials, such as the inaccuracy of the screw dislocation Peierls potential^35^ and the $\gamma$-surface problem, which are major obstacles to the prediction of the mechanical behavior of $\alpha$-Fe. Recently, the improved version of this ANN potential was used to reproduce the fracture behavior predicted by density functional theory^36^.

In the current study, we revisited the analyses of penny-shaped cracks in bcc crystals to model the cleavage fractures using the ANN Fe potential^36^ in association with the stress intensity factors of straight crack fronts. Because the adopted potential provides efficient interatomic-force calculations, it is adequate for large-scale 3D atomistic calculations. In the following sections, we show that the current atomistic approach supports the fact that {100} is the preferential cleavage plane and provides novel means to analyze cleavage and dislocation emissions from different crack fronts.

## Results

### Stress intensity factor analysis of straight crack fronts

Before analyzing curved cleavage crack fronts, the stress intensity factors (*K*s) of representative straight crack fronts were investigated; i.e., we evaluated the critical *K*s for brittle crack propagation ($K_{G}$)^19^ and for emitting a dislocation ($K_{R}$)^37^. The surface energy $\gamma _S$ and $K_{G}$ for the crack plane/front combinations are summarized in Table 1. Notice that $K_{G}$ is independent of the crack front direction and that the dependence on the crack plane is not large because $\gamma _S$ does not greatly vary with the crystal plane. The results of $K_{R}$ are also listed in Table 1 together with the values of $\theta$ and $\phi$, which are the angle between the slip plane and the crack plane and the angle between the crack front normal direction and the slip direction (i.e., Burgers vector), respectively. In contrast to $K_{G}$, $K_{R}$ varies greatly with the crack plane/front combination; for example, some $K_{R}$ values are almost equal to those of $K_{G}$, while other $K_{R}$ values greatly exceed $K_{G}$. Despite these variations, the data in Table 1 indicate that $K_{R} > K_{G}$ is satisfied for all the crack plane/front combinations; thus, the brittle responses were predicted for all the considered crack plane/front combinations.

**Table 1:** Crack-system dependence of various critical stress intensity factors (MPa $\sqrt{m}$).

Crack plane / front	$\gamma _S$ (J/$m^2$)	$K_{G}$	$\theta$ (deg)	$\phi$ (deg)	$K_{R}$	Observed response	$K_{Ic}$
(100) / [010]	2.542	1.058	45.	35.3	1.639	Brittle	1.12
(100) / [110]	2.542	1.058	90.	54.7	2.031	Brittle	1.10
(110) / [001]	2.494	1.048	90.	35.3.	1.514	Brittle	1.22
(110) / [${\bar{1}}10$]	2.494	1.048	0.	54.7	$\infty$	Brittle	1.00
(110) / [$1{\bar{1}}1$]	2.494	1.048	60.	19.5	1.276	Brittle	1.02
(111) / [${\bar{1}}10$]	2.742	1.099	35.3	54.7	1.477	Brittle	1.20
(111) / [$11{\bar{2}}$]	2.742	1.099	90.	0.	1.296	Brittle	1.16

See the main text for the details.

**Figure 2:**
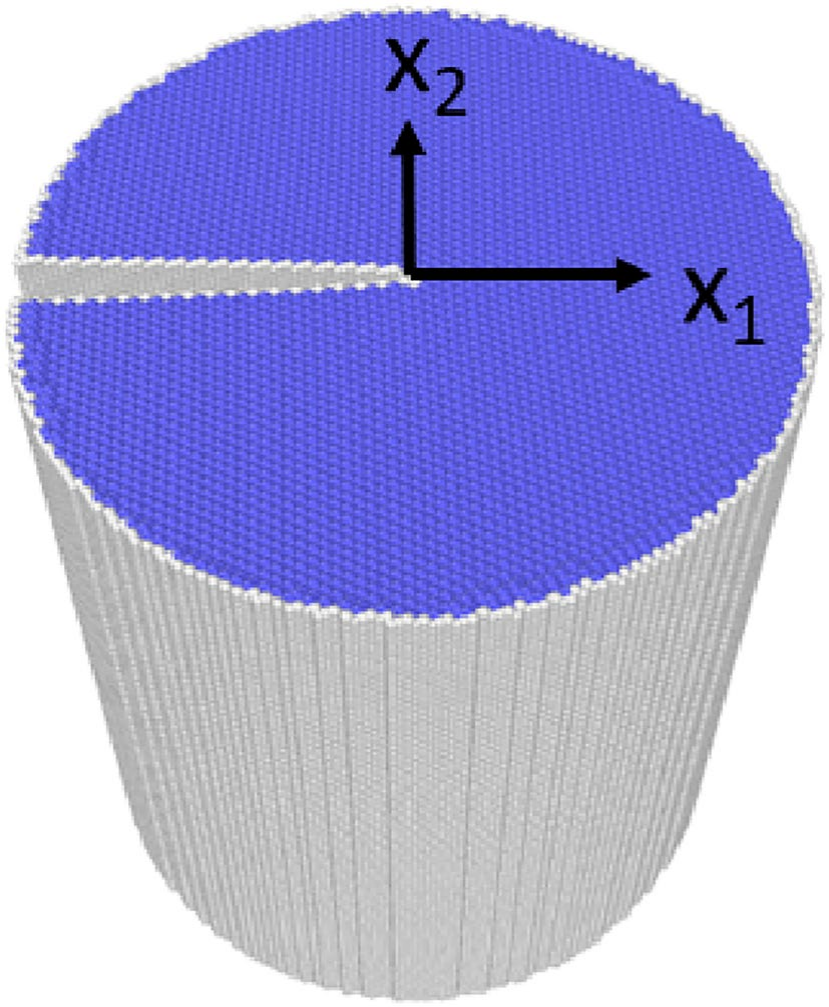
Cylindrical simulation space for the stress intensity factor analysis, where the radius of the cylinder is 130 Å, and the height of the cylinder is $\sim$300 Å. In the figure, the system is fully relaxed at $K_{I}=1.0$ MPa$\sqrt{m}$; the color is assigned by the common neighbor analysis.

The response of straight crack fronts can also be tested by atomistic simulations of a K-controlled displacement field^38^, as shown in Fig. 2. For these simulations, a cylindrical simulation space was created, and a displacement field as a function of *K* (see “Method” section for the function form) was applied to the whole space to create the sharp crack front. Notice that no plastic deformation is seen in Fig. 2, indicating that the adopted interatomic potential successfully suppresses the fictional plasticity at the crack tip. The critical stress intensity factor $K_{Ic}$ was searched in increments of $\Delta K=0.02$ MPa$\sqrt{m}$ until the crack front started propagating (i.e., a brittle fracture) or emitted a dislocation (i.e., plastic deformation). The test results are listed in Table 1. Clear brittle fractures were observed for all the considered crack plane/front combinations; i.e., no plastic deformation such as shown in Fig. 1 was observed (see Supplementary Fig. 2 to visually check various crack fronts relaxed at $K > K_{Ic}$). In other words, the theoretical analysis based on the comparison between $K_{G}$ and $K_{R}$ was completely supported by the numerical analyses. $K_{Ic}$ for each crack plane/front combination is also listed in Table 1, and the values are in the range of 1.0–1.22 J/m$^2$, which is in good agreement with the range of $K_{G}$.

To recapitulate the above discussion, all the considered straight crack fronts do not cause plastic deformation, and brittle responses are predicted. Noted that similar results were obtained for a Mo potential developed by the ANN technique^33^; i.e., the brittle fracture of all the considered crack plane/front combinations were predicted for another bcc transition metal. Nevertheless, as mentioned above, such analyses cannot successfully model the basic fracture property, that is, {100} being the naturally selected preferential cleavage plane.

### Mode-I loading of penny-shaped cleavage cracks

**Figure 3:**
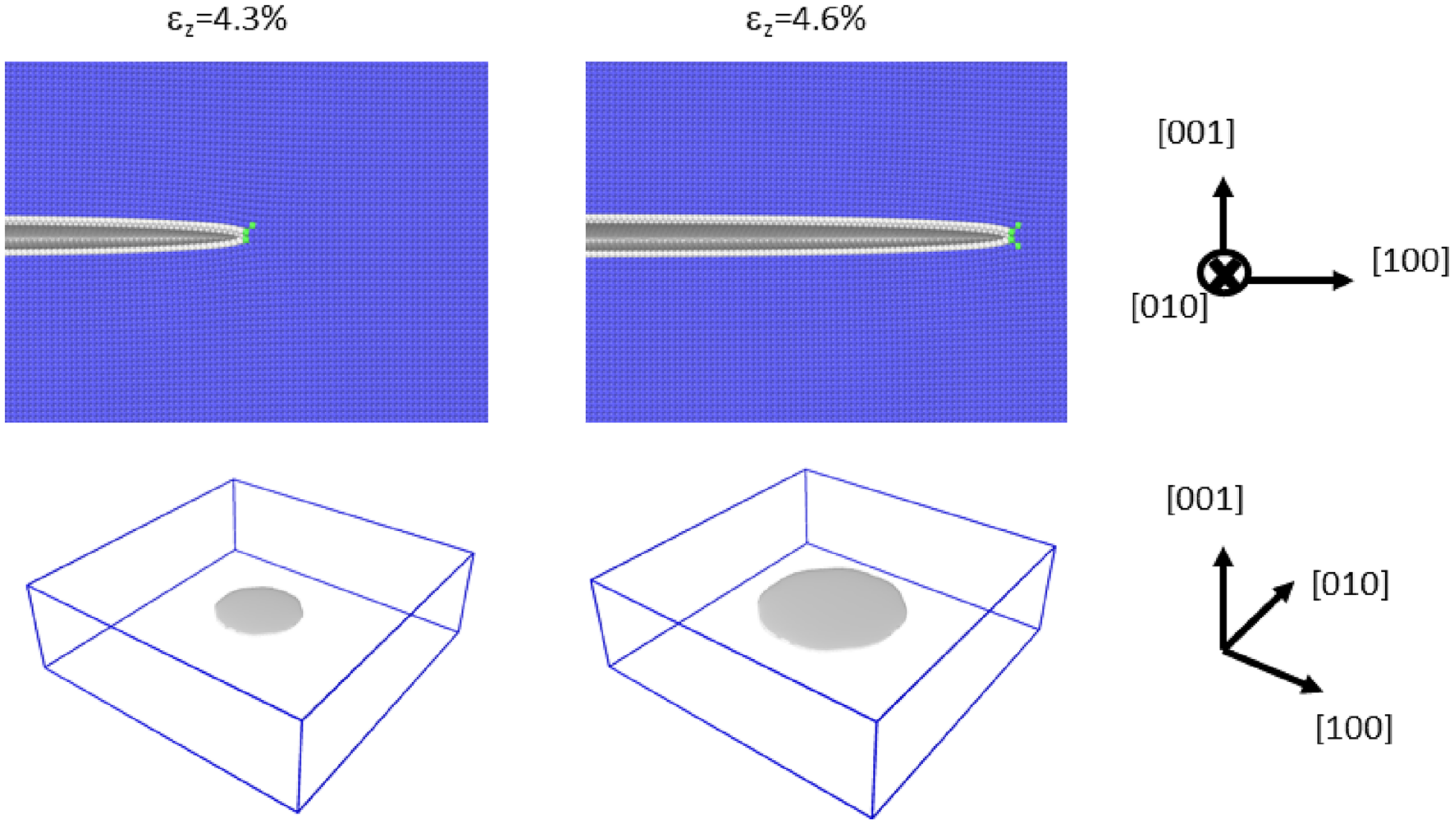
The initial condition of the {100} cleavage simulation and the snapshot after imposing 0.3% mode-I tensile strain. The upper diagrams show the cross sections in the vicinity of the crack front. The common neighbor analysis was used for determining the atomic color. The bottom diagrams show the cracked surfaces in grey.

**Figure 4:**
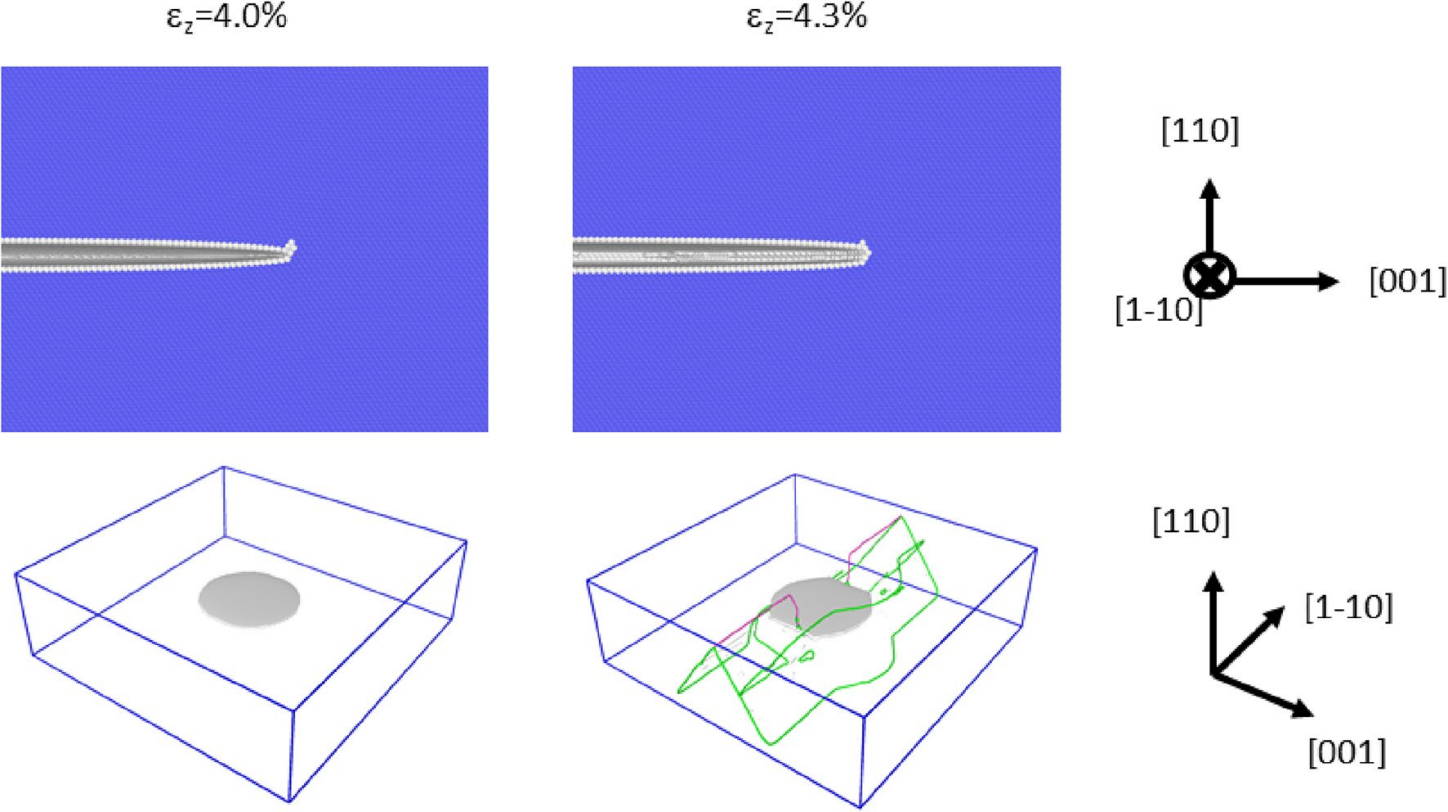
The initial condition of the {110} cleavage simulation and the snapshot after imposing 0.3% mode-I tensile strain. The upper diagrams show the cross sections in the vicinity of the crack front. The common neighbor analysis was used for determining the atomic color. The bottom diagrams show the cracked surfaces (in grey) and dislocations emitted from the crack front. The green and red lines denote 1/2<111> and <100> dislocations, respectively. Although some dislocations exist in the bottom right diagram, no lattice defect is seen in the top right diagram, because the top diagrams are magnified and shows only the vicinity of the crack tip.

**Figure 5:**
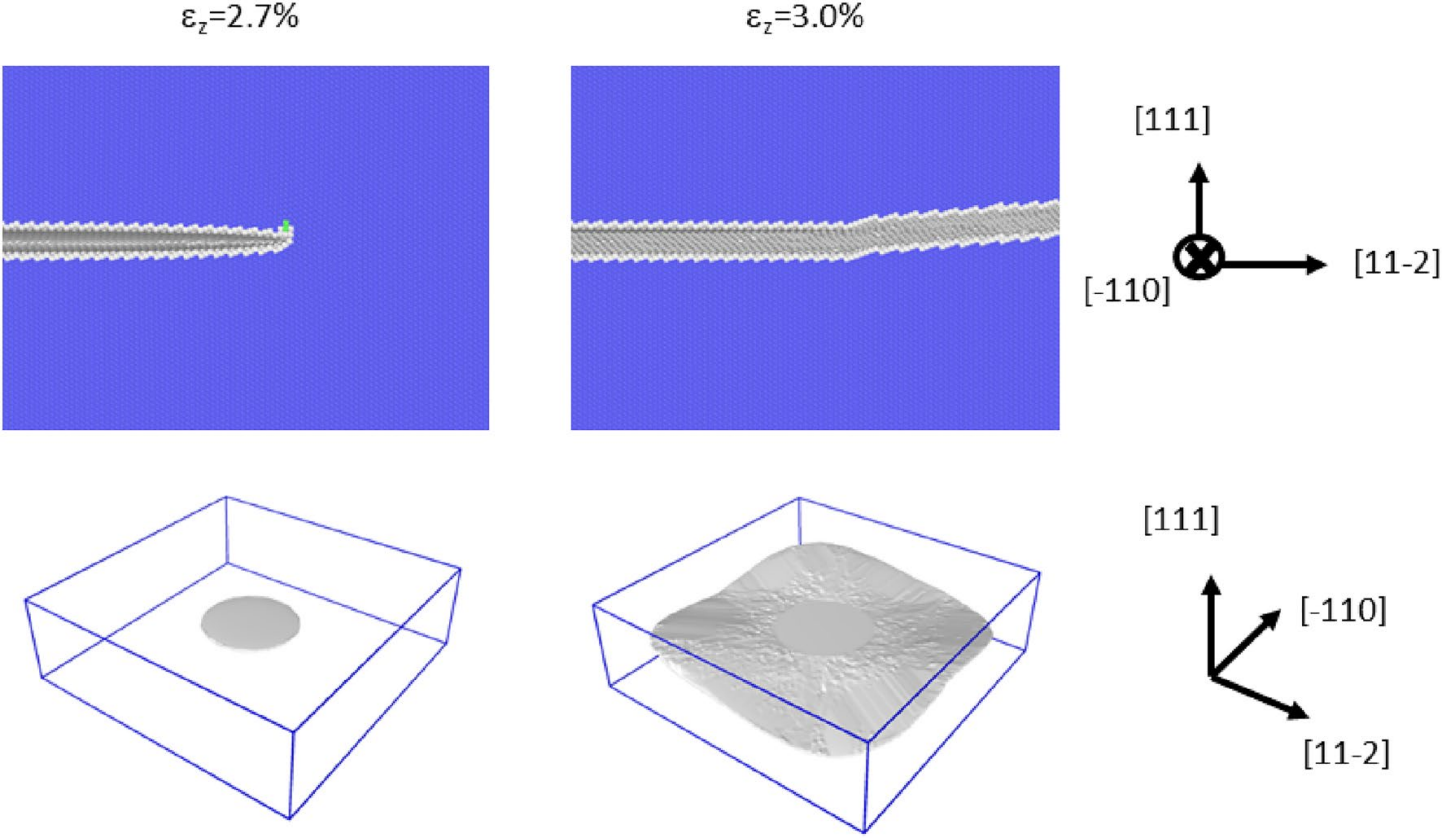
The initial condition of the {111} cleavage simulation and the snapshot after imposing 0.3% mode-I tensile strain. The upper diagrams show the cross sections in the vicinity of the crack front. The common neighbor analysis is used for determining the atomic color. The bottom diagrams show the cracked surfaces in grey.

Instead of straight crack fronts, this subsection discusses the atomistic simulations of curved crack fronts, in which the mode-I loading of penny-shaped cleavage cracks along {100}, {110}, and {111} were investigated. We conducted each simulation in a box of dimensions of 1000 $\times$ 1000 $\times$ 300 Å, wherein we inserted a penny-shaped crack of radius 200 Å. The initial conditions for the {100}, {110}, and {111} crack planes are shown in the diagrams on the left side in Figs. 3, 4 and 5, respectively. Note that tensile strain normal to the crack plane $\varepsilon _{z}$ was already imposed at the initial state to stabilize the crack. This initial strain is called $\varepsilon ^c_{z}$ in the following discussion, and it was determined by try and error. The obtained values were 0.043, 0.040 and 0.027 for {100}, {110}, and {111}, respectively. From each initial condition, we conducted mode-I loading at 0 *K* until the response of each penny-shaped crack was clearly determined. The calculation snapshots at $\varepsilon ^c_{z}$ + 0.3% are shown in the diagrams on the right side in Figs. 3 , 4 and 5. See also, Supplementary Movies 1–3 for the development of the cleavage crack during the deformation.

Figure 3 shows the development of penny-shaped cleavages along {100}. As mentioned above, the left-side diagrams show the state after the sharp crack was inserted and relaxed, i.e., the initial state. The right-side diagrams indicate the snapshot after imposing additional 0.3% mode-I loading (i.e., $\varepsilon _z=4.6\%$). The upper diagrams show the cross sections in the vicinity of the crack front. For the bottom diagrams, the dislocation identifier^39^ was applied, and no dislocation was observed. We further checked dislocation emissions up to $\varepsilon _z=5.3\%$, but no dislocation was observed; i.e., idealistic brittle behavior was always observed for the penny-shaped crack on {100}. In the previous studies^18,40,41^ in which EAM potential was applied, however, some plastic deformation such as shown in Fig. 1 was observed. Möller et al.^42^ claimed that such plastic deformation events at the cleavage crack along {100} are artifacts due to the inaccurate $\gamma$-surface curve as shown in Supplementary Fig. 1, which results in the crack fronts emitting erroneous partial dislocations. The ANN potential used in the current study successfully overcomes this problem^34^ and suppresses the emission of the partial dislocations; consequently, the plastic deformation originating from erroneous partial dislocations is considered to be suppressed in the current study.

**Figure 6:**
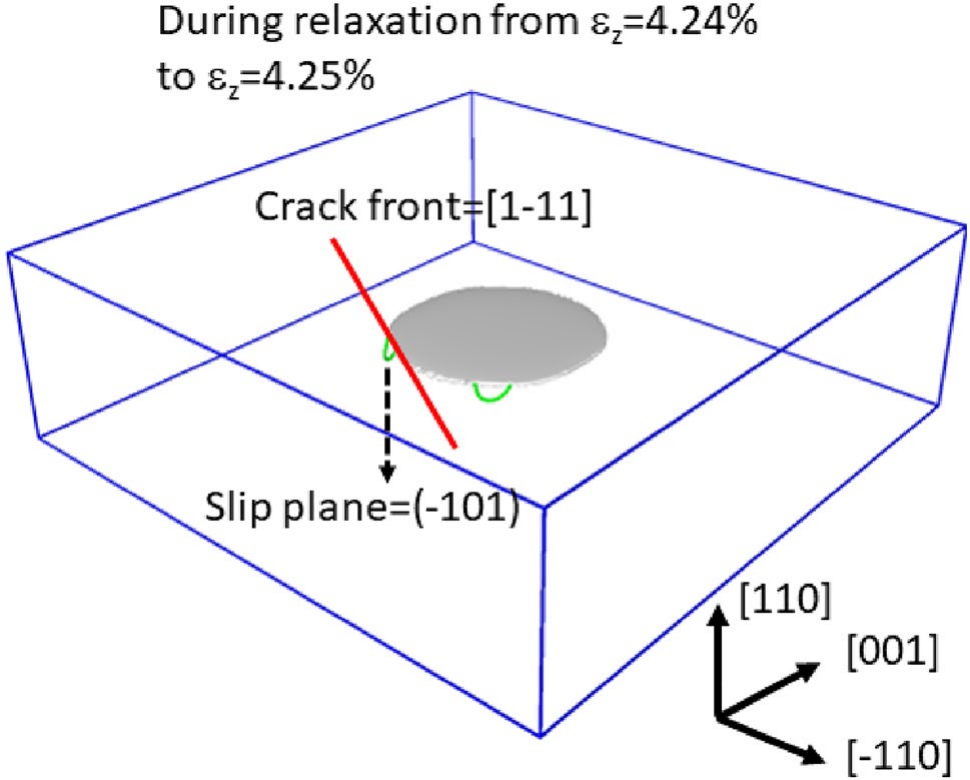
Snapshot depicting the situation immediately after dislocation emission from the crack along {110}; the gray part denotes the cracked surfaces, and the green lines are 1/2<111> dislocations; the red straight line indicates the crack front direction from which a 1/2<111> dislocation is emitted.

Figure 4 shows the diagrams for the cleavage simulation on {110}: panels are arranged similar to those in Fig. 3. In this case, the dislocations were emitted from the crack front, and the crack hardly propagated. The result in Fig. 4 supports the experimental fact that bcc transition metals such as $\alpha$-Fe do not cleave along {110} despite the lower surface energy of this plane compared to that of {100}, which is the actual cleavage plane. Our previous study^18^ based on the EAM potential had led to the same conclusion, and the current study reconfirmed this finding with the correct interatomic potential. Thus, we confidently believe that the plastic deformation in the vicinity of the curved crack front suppress the cleavage along {110}. Figure 6 shows a snapshot of the cleavage simulation immediately after the dislocation emissions. As shown in the figure, a dislocation is emitted from a crack front part parallel to the <111> direction. We confirmed that two more dislocations were emitted and that they were also emitted from the crack front part parallel to the <111> direction (See Supplementary Movie 4 to see all the emitted dislocations). The observed slip plane and direction were always {110} and <111>, respectively, as assumed in the analyses of the straight crack fronts above. This crack plane/front combination was analyzed using the stress intensity factor (see the fifth case listed in Table 1 and Supplementary Fig. 2). Note that $K_{R}$ is 1.276 MPa$\sqrt{m}$, which is the smallest finding among the considered cases, indicating that the dislocation emission is the easiest among the cases listed in Table 1. This finding suggests that dislocation emission is possible from curved crack fronts if the difference between $K_{G}$ and $K_{R}$ is small. The results shown in Fig. 4 may be connected to so-called opening softening; i.e., the unstable stacking fault energy ($\gamma _{us}$) of the slip system decreases when tensile strain is applied. In fact, $\gamma _{us}$ does decrease significantly under tensile strain (see Fig. 4 of Supplementary information of the previous study^34^). However, it is curious that only curved crack fronts can be ductile. More detailed discussion on how the curved crack front emits dislocations is given later in “Discussion”.

Figure 5 shows the diagrams for the cleavage simulation for {111}; the panels are arranged similar to those in Fig. 3. In this case, a brittle response was observed as in the case of {100}. This result suggests that {111} could be another cleavage plane of $\alpha$-Fe, but this suggestion is contradicted by experimental observations. We can speculate some possible explanations such that cleavage along the {111} plane is naturally prohibited because of the high surface energy (See Table 1) or that some plastic modes, which are not manifested in the simulation, exist.

In the previous section, we described the historical dispute regarding the selection of the preferential cleavage plane. The results reported herein support the idea that plasticity in the vicinity of the crack tip determines the plane. A critical suggestion is that the same conclusion cannot be attained only by analyzing straight crack fronts, because in these cases only brittle responses, as listed in Table 1, are observed.

## Discussion

**Figure 7:**
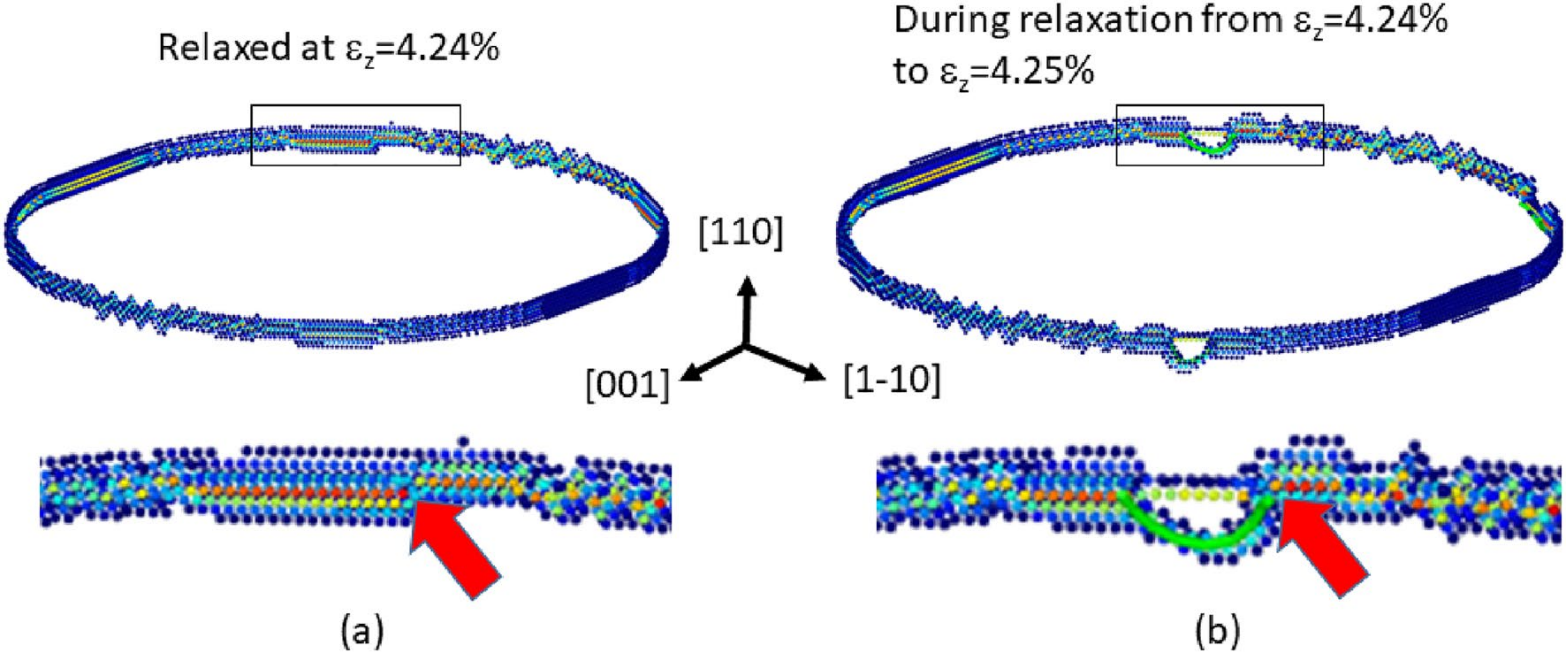
Tensile stress distributions before and after the dislocation (the green lines) emitted from the penny-shaped crack front along {110}: Only atoms with a relatively large $\sigma _{zz}$ are visible, and the color shifts towards red as $\sigma _{zz}$ increases, where the z direction is [110]. (**a**) The relaxed state at $\varepsilon _{z}=4.24\%$; (**b**) a snapshot in the course of relaxation from $\varepsilon _{z}=4.24\%$ to $\varepsilon _{z}=4.25\%$. Each bottom diagram shows the magnified view in the area bounded by the rectangle of the upper diagram.

The previous section showed that a certain curved crack front with relatively small $K_{R}$ close to $K_{G}$ can emit dislocations even though their idealistic straight crack fronts are theoretically brittle. Such a situation was observed in the case of the crack systems of {110}/<111>. Because this mechanism is unclear, it is discussed more detail here. As seen in Fig. 7, we analyzed the stress distribution before and after the dislocation emission from the crack of {110}/<111>. In the figure, only the atoms with relatively large tensile stress perpendicular to the crack plane are shown. Because the stress is intensified in the vicinity of the crack tip, the ring shape appears by this filtering. The red atoms have the largest stress and indicate the exact location of the crack tip. The lower diagrams show the magnified views of the crack front in the <111> direction. Note that the series of red atoms are not aligned straight but exhibit a step (or a jog) as indicated by the arrows. As seen in Fig. 7b, this is the exact location where the slip motion (i.e., the dislocation emission) is initiated. We concurrently witnessed two more dislocation emission events (see Supplementary Movie 4) and confirmed that the jogs at the <111> crack front part were triggering points also for these two slip events. Because the idealistic straight crack fronts discussed in the stress intensity factor analyses do not have such jogs, we think that this triggering point, which is specific to the curved {110}/<111> crack system, decreased the local $K_{R}$ (or increased the local $K_{G}$). Hence, what we learned here is that a crack system whose $K_{R}$ is relatively small can emit dislocations via some disturbances, even though the idealistic straight crack front satisfies $K_{G} < K_{R}$.

Regarding the cleavage along {100}, it is difficult for any disturbances to reverse this relationship and lead to dislocation emissions because $K_{R}$ for the crack fronts of {100} cleavage greatly exceeds the $K_{G}$ values, as seen from Table 1. Conceivably, this finding can explain the experimental fact that {100} plane is the preferential cleavage plane of $\alpha$-Fe crystals.

In summary, the current study highlights the importance of large-scale 3D atomistic modeling for correctly predicting the macroscopic property because simple straight crack fronts, which are free from natural disturbances (or randomness), may not necessarily reflect the realistic situation. It also highlights the importance of machine-learning techniques for producing highly accurate empirical potentials because the conventional EAM potentials often cause fictional behaviors in the vicinity of crack fronts, possibly leading to a wrong conclusion regarding the crack-tip plasticity.

## Methods

### Stress intensity factor analyses

$K_{G}$ is calculated as follows:

1 $$\begin{aligned} K_{G} = \sqrt{\frac{2E\gamma _S}{1-\nu ^2}} \end{aligned}$$

where $\gamma _S$ is the surface energy of each plane. The $\gamma _S$ value was evaluated for the adopted empirical potential within the current study by measuring the excess energy when numerically breaking a small crystal (e.g., 48 Å$\times$ 48 Å$\times$ 60 Å) along the surface. *E* is the Young’s modulus (204 GPa), and $\nu$ is the Poisson’s Ratio (0.27). To evaluate $K_{R}$^37^, we assumed that the slip system is <111>{110}; i.e., the slip direction and plane are <111> and {110}, respectively. $K_{R}$ is calculated as follows:

2 $$\begin{aligned} K_{R} = (\cos ^2{(\theta /2)} \sin {(\theta /2)})^{-1} \sqrt{\frac{2\mu }{1-\nu }\gamma _{us}(1+(1-\nu )\tan ^2{\phi }} \end{aligned}$$

where $\mu$ is the shear modulus (78 GPa); $\gamma _{us}$ is the unstable stacking fault energy of the slip system of <111>{110} (0.982 J/m$^{2}$). The $\gamma _{us}$ value was evaluated for the adopted empirical potential within the current study by calculating the generalized stacking fault energy curve^42^ (see Fig. 2a of the reference).

To numerically test brittleness (or plasticity) a specified straight crack front, we first created a cylindrical simulation space filled with $\alpha$-Fe crystal. The periodic boundary condition was applied in the direction of the height; i.e., the two bases were connected to each other. After the volume relaxation using the adopted interatomic potential, we applied the following K-controlled displacement field^38^ to each atom in the system:

3 $$\begin{aligned} u_1 = \frac{K_I}{\mu }\sqrt{\frac{r}{2\pi }}\cos \frac{\beta }{2} \left( 1-2\nu +\sin ^2\frac{\beta }{2} \right) , \end{aligned}$$

4 $$\begin{aligned} u_2 = \frac{K_I}{\mu }\sqrt{\frac{r}{2\pi }}\sin \frac{\beta }{2} \left( 2-2\nu -\cos ^2\frac{\beta }{2} \right) \end{aligned}$$

where $K_I$ is the mode-I stress intensity factor, and *r* and $\beta$ are defined as follows:

5 $$\begin{aligned} r=\sqrt{x_1^2+x_2^2}, \end{aligned}$$

6 $$\begin{aligned} \beta = \tan ^{-1} (x_2/x_1), \end{aligned}$$

where $x_1$ and $x_2$ are the axes normal to the crack front direction, as seen in Fig. 2. After applying the above displacement field, each atomic position was relaxed by the interatomic potential with a fixed outer shell, whose thickness is 5 Å. As seen in Table 1, several cases of the crack front were tested in the current study. In each case, we first applied the displacement field corresponding to $K_I \le 1.0$. Subsequently, we gradually increased $K_I$ in increments of $\Delta K_I=0.02$ MPa $\sqrt{m}$ until either brittle or plastic response was observed. The $K_{Ic}$ values in Table 1 are the smallest values that resulted in the initiation of some recognizable changes at the crack tip.

### Molecular statics simulation of penny-shaped cleavage cracks

A perfect $\alpha$-Fe crystal of size $L_x \times L_y \times L_z$ was numerically created. In the simulation, the z plane was considered as the cleavage plane. We adopted $L_x = L_y = 1000$ Å, and $L_z = 300$ Å; this is almost the minimum size required for such a simulation^18^. Then, the simulation box was strained in the mode-I direction up to a certain tensile strain value of $\varepsilon ^c_z$, and a sharp penny-shaped crack, with a radius of 200 Å, was inserted. We chose $\varepsilon ^c_z$ by try and error so that the inserted crack neither advanced nor regressed and so that the curved crack front did not cause any plastic deformation. To be more specific, we created a penny-shaped vacuum space perpendicular to the z-direction at the center of the simulation box by displacing a group of atoms. To generate a sharp crack front, we applied trigonometric functions and relaxed the whole system using the applied empirical potential at 0 K. To observe crack propagation, we conducted the mode-I loading simulation at 0 K as follows: In each step of deformation, tensile strain was added in increments of $\Delta \varepsilon _{z}=0.01\%$ with compressive deformation along the x- and y-directions considering the Poisson’s ratio (0.27) for $\alpha$-Fe; subsequently, the system was relaxed under a fixed boundary condition for all three directions. Note that the molecular dynamics simulations at finite temperatures were too cumbersome to be applied to the current large-scale (over 20 million atoms) simulations.

### Applied software systems

Throughout this study, we used the LAMMPS code^43^ for the atomistic simulations and OVITO^44^ to visualize the results along with a dislocation analysis tool (DXA)^39^ available in OVITO. OVITO was also used to determine key orientations such as the habit planes of emitted dislocations. We applied a Fe empirical potential obtained by a machine-learning technique^34,36^. The ANN potential was applied through a module implemented in LAMMPS, namely, modified Atomic Energy Network (ænet)^45^ for the LAMMPS library.

## Supplementary Information

Supplementary Figure 1.

Supplementary Figure 2.

Supplementary Video 1.

Supplementary Video 2.

Supplementary Video 3.

Supplementary Video 4.

Supplementary Legends.

## Data Availability

All the numerical data that support the findings of this study are available from the corresponding author upon reasonable request. The parameter file of the potential adopted here is freely available online from https://github.com/HidekiMori-CIT/aenet-lammps.
