# Supplementary material for: Cleavages along {110} in bcc iron emit dislocations from the curved crack fronts
Source: Sci Rep. 2022 Nov 16;12:19701. doi: 10.1038/s41598-022-24357-5 (PMC9668986; doi:10.1038/s41598-022-24357-5)
Supplement: Supplementary file 1 — Supplementary Figure 1. [file 41598_2022_24357_MOESM1_ESM.pdf]

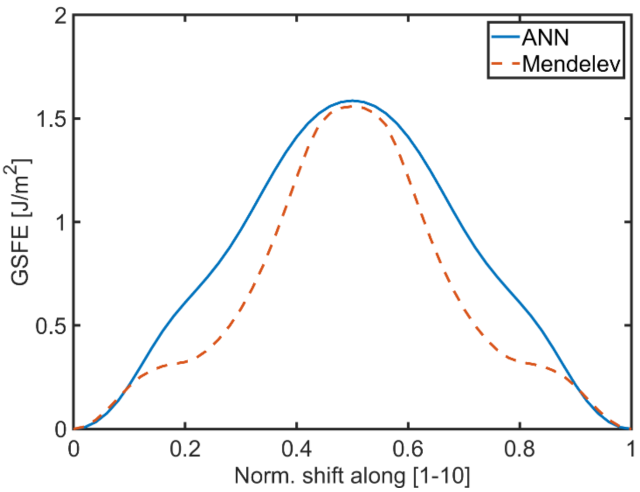

Supplementary Figure 1:
General stacking fault energy (GSFE) or γ-surface of the (110) glide plane in [1-10] direction under 5% external equi-biaxial strain in [110] and [1-10] directions. Compared with the ANN potential, the result for Mendelev potential [1], a common EAM potential, have "depressions", which are not observed from DFT analyses [2]. Such depressions are considered to be an origin of the fictional plastic deformation in the vicinity of the crack tips when EAM potentials are applied to fracture analyses.

References

[1] M. I. Mendelev, S. Han, D. J. Srolovitz, G. J. Ackland, D. Y. Sun, and M. Asta, Philos. Mag. **83**, 3977 (2003).

[2] J. J. Möller, M. Mrovec, I. Bleskov, J. Neugebauer, T. Hammerschmidt, R. Drautz, C. Elssäser, T. Hickel, and E. Bitzek, Phys. Rev. Materials **2**, 093606 (2018).
