# Supplementary material for: Cleavages along {110} in bcc iron emit dislocations from the curved crack fronts
Source: Sci Rep. 2022 Nov 16;12:19701. doi: 10.1038/s41598-022-24357-5 (PMC9668986; doi:10.1038/s41598-022-24357-5)
Supplement: Supplementary file 2 — Supplementary Figure 2. [file 41598_2022_24357_MOESM2_ESM.pdf]

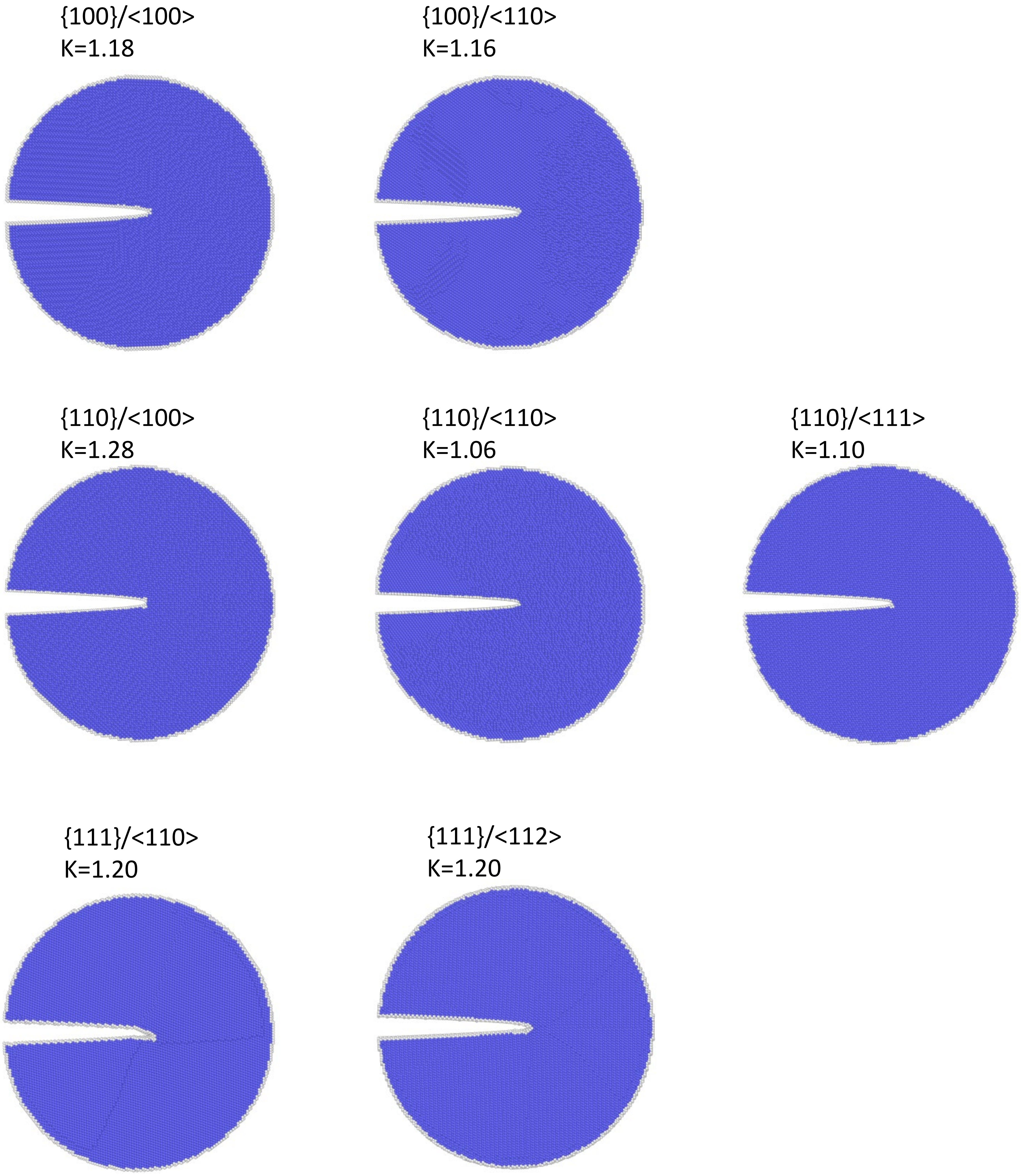

Supplementary Figure 2:
Numerical simulation results from stress intensity factor analyses for various crack systems. Each K value (MPa $\sqrt{m}$) is slightly larger than its critical value (K_{Ic}); thus, it is possible to judge whether each crack front is brittle or plastic. The common neighbor analysis and dislocation analysis are used to detect plastic deformation. All of these straight crack fronts are determined to be brittle, because no plastic deformation is observed.
