# Supplementary material for: Cleavages along {110} in bcc iron emit dislocations from the curved crack fronts
Source: Sci Rep. 2022 Nov 16;12:19701. doi: 10.1038/s41598-022-24357-5 (PMC9668986; doi:10.1038/s41598-022-24357-5)
Supplement: Supplementary file 7 — Supplementary Legends. [file 41598_2022_24357_MOESM7_ESM.doc]

Supplementary Movie 1

Development of penny-shaped cleavages along the {100} plane during the mode-I loading: The cracked surface is colored in grey.

Supplementary Movie 2

Development of penny-shaped cleavages along the {110} plane during the mode-I loading: The cracked surface is colored in grey; the green and red lines denote 1/2<111> and <100> dislocations, respectively.

Supplementary Movie 3

Development of penny-shaped cleavages along the {111} plane during the mode-I loading: The cracked surface is colored in grey.

Supplementary Movie 4

Rotating image of the dislocation emission from the crack along {110}; the gray part denotes the cracked surfaces, and the green lines are 1/2<111> dislocations.
